# Supplementary material for: Mouse Mammary Tumour Virus (MMTV) in Human Breast Cancer—The Value of Bradford Hill Criteria
Source: Viruses. 2022 Mar 30;14(4):721. doi: 10.3390/v14040721 (PMC9028876; doi:10.3390/v14040721)
Supplement: Supplementary file 1 [file viruses-14-00721-s001.zip › viruses-1607698-supplementary.pdf]

Supplementary Table S1. Studies of MMTV in human breast cancer with negative outcomes.

Study	Country	Method	MMTV Breast Cancer Identification
Zangen 2002 [50]	Italy	PCR	0/18
Witt 2003 [51]	Austria	PCR	0/50
Mant 2004 [52]	United Kingdom	PCR	0/44
Bindra 2007 [53]	Sweden	PCR	0/18
Frank 2008 [54]	Germany	Hybridisation	0/23
Fukuoka 2008 [55]	Japan	PCR/hybridisation	0/46
Park 2011 [3]	Australia	PCR	0/42
Motamedifar 2012 [56]	Iran	PCR	0/50
Tabriz 2013 [57]	Iran	PCR	0/40
Morales-Sanchez 2013 [58]	Mexico	PCR	0/65
Perzova 2017 [4]	USA	PCR	1/10 (contaminated author assessment)
